# Model-based analysis of subthreshold mechanisms of spinal cord stimulation for pain

**DOI:** 10.1088/1741-2552/ad0858

**Published:** 2023-11-09

**Authors:** Evan R Rogers, Ehsan Mirzakhalili, Scott F Lempka

**Affiliations:** 1 Department of Biomedical Engineering, University of Michigan, Ann Arbor, MI, United States of America; 2 Biointerfaces Institute, University of Michigan, Ann Arbor, MI, United States of America; 3 Department of Anesthesiology, University of Michigan, Ann Arbor, MI, United States of America

**Keywords:** spinal cord stimulation, chronic pain, computer simulation, neuromodulation, electric stimulation

## Abstract

*Objective.* Spinal cord stimulation (SCS) is a common treatment for chronic pain. For decades, SCS maximized overlap between stimulation-induced paresthesias and the patient’s painful areas. Recently developed SCS paradigms relieve pain at sub-perceptible amplitudes, yet little is known about the neural response to these new waveforms or their analgesic mechanisms of action. Therefore, in this study, we investigated the neural response to multiple forms of paresthesia-free SCS. *Approach.* We used computational modeling to investigate the neurophysiological effects and the plausibility of commonly proposed mechanisms of three paresthesia-free SCS paradigms: burst, 1 kHz, and 10 kHz SCS. Specifically, in C- and A*β*-fibers, we investigated the effects of different SCS waveforms on spike timing and activation thresholds, as well as how stochastic ion channel gating affects the response of dorsal column axons. Finally, we characterized membrane polarization of superficial dorsal horn neurons. *Main results.* We found that none of the SCS waveforms activate nor modulate spike timing in C-fibers. Spike timing was modulated in A*β*-fibers only at suprathreshold amplitudes. Ion channel stochasticity had little effect on A*β*-fiber activation thresholds but produced heterogeneous spike timings at suprathreshold amplitudes. Finally, local cells were preferentially polarized in their axon terminals, and the magnitude of this polarization was dependent on cellular morphology and position relative to the stimulation electrodes. *Significance.* Overall, the mechanisms of action of subparesthetic SCS remain unclear. Our results suggest that no SCS waveforms directly activate C-fibers, and modulation of spike timing is unlikely at subthreshold amplitudes. We conclude that potential subthreshold neuromodulatory effects of SCS on local cells are likely to be presynaptic in nature, as axons are preferentially depolarized during SCS.

## 1. Introduction

Spinal cord stimulation (SCS) is a neurostimulation treatment for chronic pain that is refractory to conventional treatment options (e.g. pharmaceuticals, physical therapy). One or more multi-contact electrode arrays are implanted into the posterior epidural space within the vertebral column and pulses of mild electrical current are applied to the spinal cord. SCS was originally conceived to exploit the ‘gate control theory of pain’, which suggests that activating large-diameter A*β*-fibers (mechanoreceptors carrying innocuous touch information in the dorsal column (DC) white matter pathway) will inhibit transmission of painful signals to the brain [1, 2]. Importantly, the SCS-induced action potentials in these fibers also travel orthodromically towards the brainstem, generating a buzzing or tingling percept (i.e. paresthesia) [3]. Maximizing pain-paresthesia spatial overlap is associated with successful SCS outcomes [3–5].

Unfortunately, this standard SCS approach possesses several limitations. Many patients find the paresthetic sensations intolerable or distracting, and paresthesia-related complications are major reasons for device explantations [6, 7]. Furthermore, these paresthesias can interfere with daily functions, such as sleeping and driving [8], and postural changes induce potentially uncomfortable fluctuations in sensation as the spinal cord moves relative to the implanted electrodes [9, 10]. These concerns can be partially mitigated by closed-loop systems which automatically adjust stimulation parameters in response to postural changes to avoid under- or overstimulation [11].

Recently, several novel SCS paradigms have been developed that produce pain relief at subparesthetic amplitudes (i.e. below the amplitude necessary to generate perceivable paresthesia), and many patients express a preference for paresthesia-free pain relief [12, 13]. Notable examples include burst and kilohertz-frequency SCS [12, 14]. Both burst and 10 kHz SCS (a common implementation of kilohertz-frequency SCS) have been rapidly adopted in the clinic following successful pivotal trials demonstrating superior results compared to conventional stimulation [15, 16]. While less common clinically, 1 kHz SCS has been shown to produce similar pain relief to 10 kHz SCS, while importantly using less energy (extending battery life) and not requiring proprietary hardware (thereby expanding access to more patients) [17]. There is a consensus, based on clinical observations and preclinical evidence, that these novel waveforms likely engage different pain-relieving mechanisms compared to conventional paresthesia-based SCS [14]. However, the specific mechanisms underlying these approaches remain largely speculative and are actively debated.

To date, many hypotheses have been proposed regarding the neural effects of these paresthesia-free stimulation waveforms [12, 14]. Still, these suppositions remain largely theoretical and untested. In addition, these proposed mechanisms typically lack a biophysical basis for how these effects are achieved. Two of the most popular working hypotheses for 10 kHz and burst SCS are selective activation of dorsal horn inhibitory interneurons and modulation of the medial pain pathway (typically proposed to occur via selective activation of anti-nociceptive C-fibers), respectively [18–20]. Evidence for these hypotheses is provided by rodent spinal recordings [18] and neuroimaging [21–24]. It is also commonly proposed that novel SCS waveforms can disrupt pathological synchronous firing or activate fibers in an asynchronous or pseudo-spontaneous manner (thereby producing pain relief without paresthesia) [20, 25, 26]. However, no available data elucidate the biophysical basis for how these effects would be achieved.

In this study, we used a computational modeling approach to investigate postulated subparesthetic mechanisms of action of SCS, which permits analyzing the biophysical effects of SCS at human scale. Specifically, we examined the following commonly proposed hypotheses: whether novel SCS waveforms desynchronize spike timing [26], if SCS selectively activates C-fibers [20], and if SCS directly modulates gray matter neurons [18, 27, 28] (e.g. increased excitability or activation due to direct membrane polarization [29]). Additionally, we considered the effects of stochastic ion channel behavior in the response of DC fiber models to SCS to investigate whether these properties will reduce thresholds or produce asynchronous activation [30, 31]. For these analyses, we evaluated the response to conventional (50 Hz), burst, 1 kHz, and 10 kHz SCS.

Overall, our results suggest C-fibers are not activated nor desynchronized by any form of stimulation. Membrane polarization of neurons within the dorsal horn was small, but strongest in their axon terminals, suggesting any local dorsal horn effects are likely to be presynaptic in nature. Finally, including stochastic ion channel properties had a negligible effect on activation thresholds but did alter suprathreshold firing patterns during all SCS waveforms. Overall, these results help elucidate the neural response to several clinically relevant SCS waveforms.

## 2. Materials and methods

**Figure 1.**
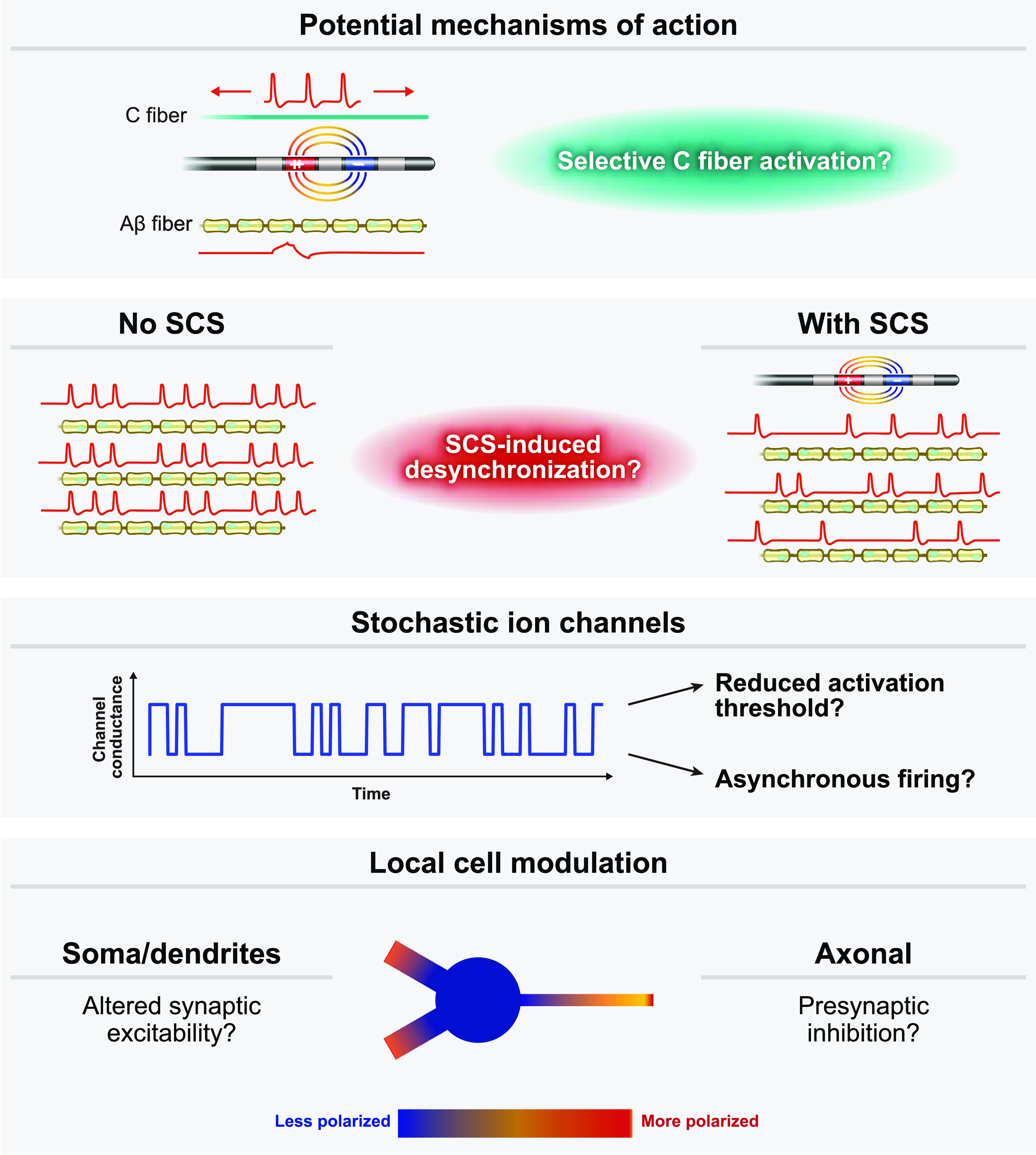
We evaluated four potential subparesthetic mechanisms of action: (1) do subparesthetic waveforms SCS selectively activate C-fibers? (2) Do subparesthetic SCS waveforms modulate spike timing and desynchronize afferent firing? (3) Do subparesthetic SCS waveforms interact synergistically with the stochastic nature of ion channel gating to reduce activation thresholds or produce asynchronous firing? (4) Do subparesthetic SCS waveforms directly modulate the excitability of neurons within the dorsal horn?

We utilized a two-stage computer modeling approach to evaluate several theoretical subparesthetic mechanisms of novel SCS technologies. First, we used the finite element method to evaluate the spatial electric potential fields generated in the spinal cord during SCS. Then, we applied these electric potentials to biophysical multi-compartment neuron models, scaled by the appropriate temporal stimulation waveform, and assessed the neural response using the NEURON computational software package [32] (v7.4) through a Python interface. We tested several potential hypotheses, including direct activation of C-fibers, action potential desynchronization, local cell modulation, and reduced activation thresholds due to stochastic ion channel properties (figure 1). Each of these sub-analyses is described in more detail below.

### 2.1. Volume conductor model

We utilized a previously developed volume conductor model of the lower thoracic spinal cord to evaluate the spatial electric potential field generated during SCS using a clinically relevant bipolar stimulation configuration (i.e. one cathode and one anode). This model is extensively described in its original publication [33]. Briefly, this model contains domains representing gray matter (electrical conductivity of 0.23 S m^−1^), anisotropic white matter (including dorsal rootlets) (0.6 S m^−1^ longitudinally, 0.083 S m^−1^ transversely), cerebrospinal fluid (1.7 S m^−1^), dura mater (0.6 S m^−1^), epidural tissue (0.25 S m^−1^), vertebral bone (0.02 S m^−1^), intervertebral disc (0.65 S m^−1^), electrode encapsulation (0.11 S m^−1^), and a general thorax domain (0.25 S m^−1^). Electrode contacts were 3 mm in length and 1.3 mm in diameter, separated by 1 mm edge-to-edge spacing. We positioned the electrode array medio-laterally over the anatomical midline. We used COMSOL Multiphysics (COMSOL, Inc., USA) to numerically evaluate the Laplace equation $\nabla \cdot \sigma \nabla V = 0$ to find the spatial electric potential field (*V*: electric potential, $\sigma $: conductivity) throughout the spinal cord. We modeled all stimulation as current-controlled, and we applied unit currents (1 mA) at active contacts, whereas we modeled the inactive contacts as equipotential surfaces with no net current and the electrode shaft as a perfect insulator. We modeled bipolar stimulation, with the cathode and anode separated by one inactive contact corresponding to a center-to-center spacing of 8 mm between the active electrodes (with the cathode positioned rostrally). We calculated the bipolar-SCS electric potential field as the superposition of results obtained for running simulations for each of the active electrodes independently. In each case, we grounded the outer domain of the volume conductor model.

### 2.2. Stimulation waveforms

**Figure 2.**
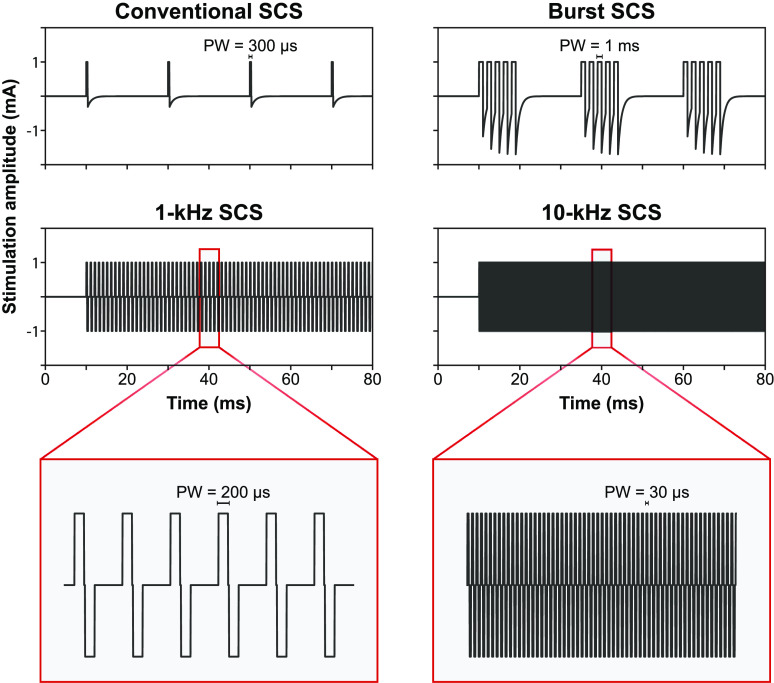
The four SCS waveforms considered in this study. Conventional SCS is delivered at an amplitude that generates paresthesia, whereas the other waveforms are typically delivered at subparesthetic amplitudes. PW: pulse width.

For each analysis, we evaluated the neural response to four clinically relevant SCS waveforms (figure 2). These stimulation paradigms were conventional, burst, 1 kHz, and 10 kHz SCS. Importantly, conventional SCS is intended to maximize overlap between paresthesia and the painful areas, whereas the three other waveforms are delivered clinically at subparesthetic amplitudes.

We modeled conventional SCS as having a 300 *μ*s stimulation pulse followed by a passive discharge phase. We delivered stimulation at 50 Hz. Burst SCS consisted of bursts of five pulses (pulse width of 1 ms) with passive discharging both between individual pulses and at the end of the burst. The intra-burst frequency was 500 Hz and the inter-burst frequency was 40 Hz [34]. For both conventional and burst SCS, we calculated the passive discharge phase using a previously developed circuit model of bipolar stimulation [35]. We modeled 1 kHz SCS as symmetric biphasic rectangular stimulation with a pulse width of 200 *μ*s (interphase interval of 80 *μ*s) delivered at a rate of 1000 Hz [36, 37]. Finally, we modeled 10 kHz SCS as symmetric biphasic rectangular stimulation, with a pulse width of 30 *μ*s (interphase interval of 20 *μ*s) delivered at a rate of 10 000 Hz [15].

### 2.3. C-fiber activation thresholds

**Figure 3.**
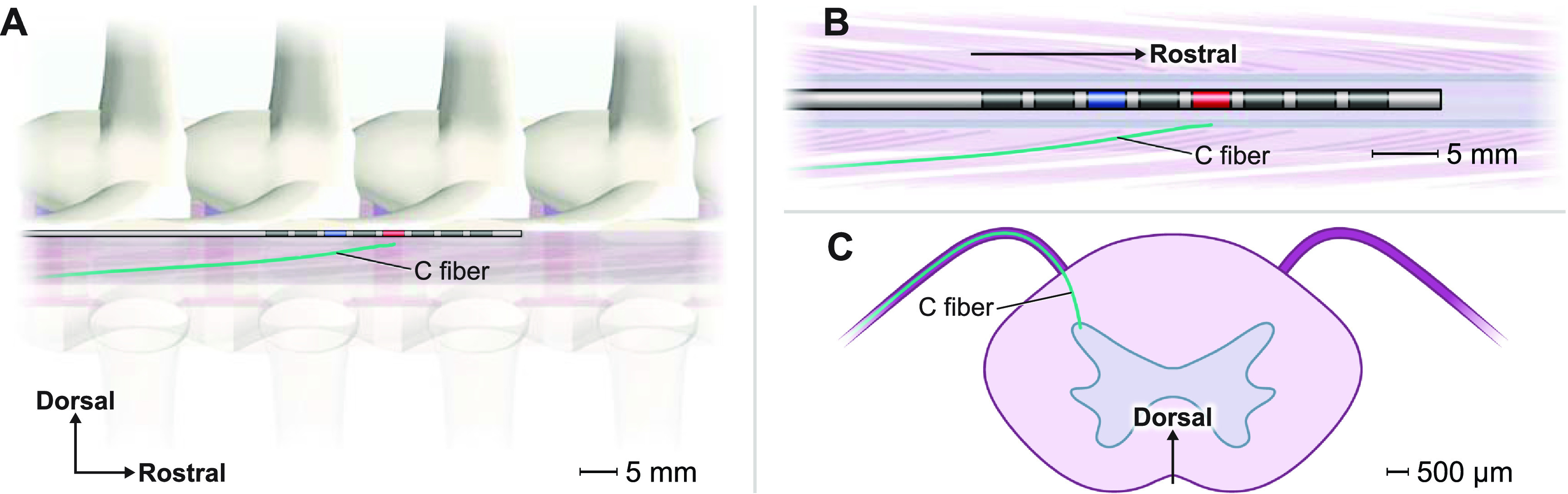
Overview of the C-fiber model. (A) Sagittal view of the C-fiber (cyan). The red electrode is the cathode, and the blue electrode is the anode. (B) Posterior view of the C-fiber ascending in a dorsal root before entering the spinal cord. (C) Axial view of the C-fiber terminating in the superficial dorsal horn.

Selective activation of C-fibers has been proposed as a mechanism for subparesthetic SCS [19, 20]. To test this hypothesis, we utilized a previously developed C-fiber model [38] and evaluated the C-fiber response to each SCS waveform. Briefly, this nonmyelinated C-fiber model contains a passive leak conductance (1 × 10^−4^ S cm^−2^) and active TTX-sensitive Na_v_1.7, TTX-resistant Na_v_1.8, and slow TTX-resistant Na_v_1.9 channels, as well as delayed rectifier and A-type potassium conductances. Additionally, the specific membrane capacitance and axial resistance are 1.0 *μ*F cm^−2^ and 100 Ω cm, respectively, with an axon diameter of 1 *μ*m. The C-fiber model ascended in the dorsal rootlets before entering the spinal cord white matter (figure 3). From here, the fiber ascended in the white matter dorsal to the gray matter boundary (Lissauer’s tract) before finally terminating in the superficial dorsal horn at the level of the cathode. We calculated activation thresholds via a binary search algorithm with a resolution of 0.1 mA. We also shifted model fibers into the rootlets caudal and rostral to the original model to evaluate sensitivity to rostro-caudal position.

### 2.4. Spike timing

**Figure 4.**
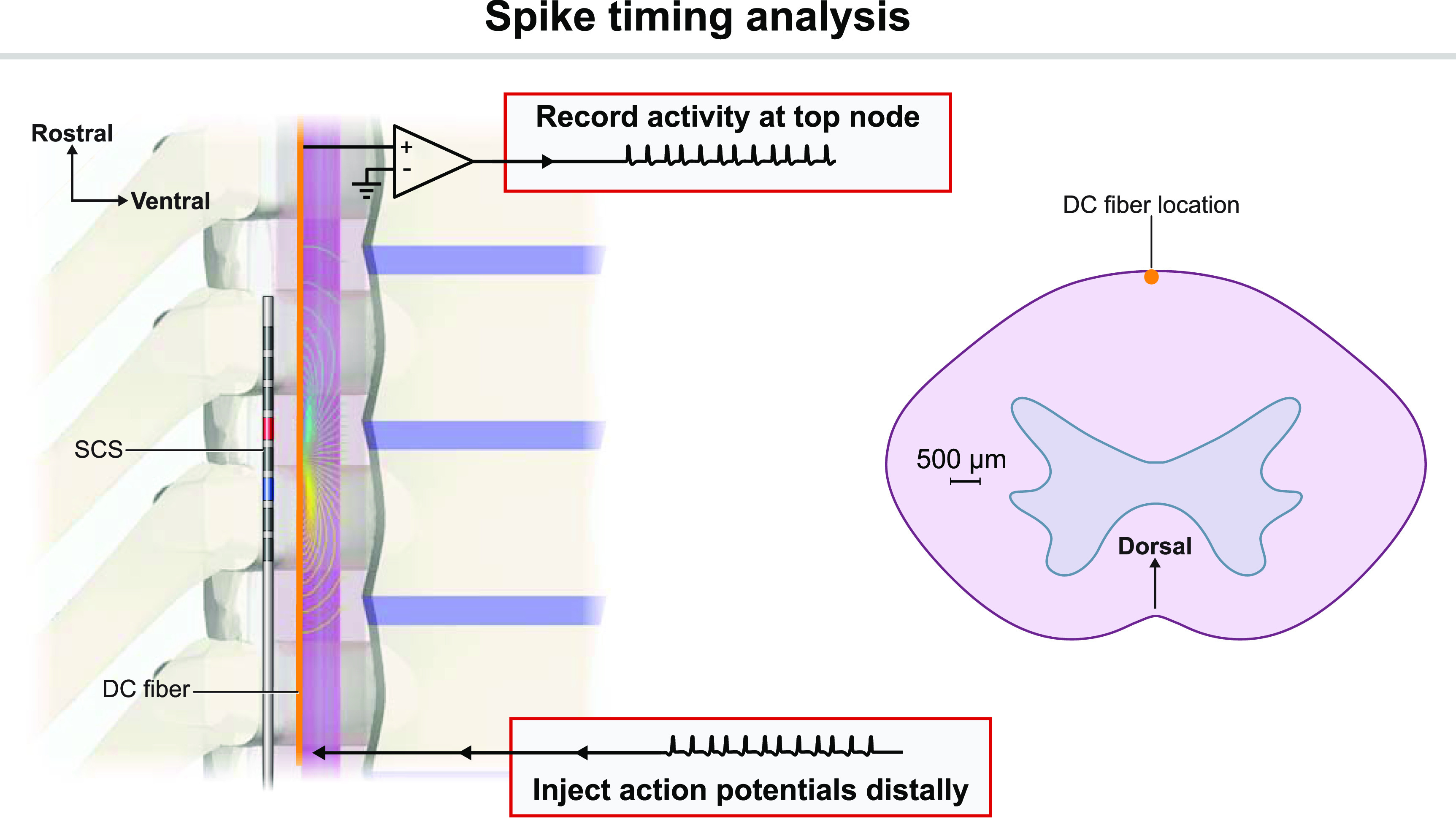
Overview of our analysis of the effects of SCS on spike timing. We injected a spike train into one end of an afferent fiber (C- or A*β*-fiber) and recorded at the terminal node of Ranvier. We used the Victor–Purpura metric to quantify differences in spike trains. On the right, we show the location of the dorsal column (DC) A*β*-fiber used for all analyses. For the spike timing analysis, the C-fibers were located in the dorsal rootlet and Lissauer’s tract, which is superficial to the dorsal horn and adjacent to the dorsal columns. The C-fiber locations are shown in figure 3.

The nerve fibers in the vicinity of the SCS electrodes transmit action potentials from the periphery into the central nervous system. Thus, one plausible mechanism of subparesthetic neurostimulation is modulating spike timing of ongoing nociceptive signaling, e.g. producing analgesia by desynchronizing firing patterns in a population of nociceptive fibers [19, 20, 26, 39]. For this reason, we investigated how the different SCS waveforms might affect spike timing in afferent fibers (figure 4).

**Figure 5.**
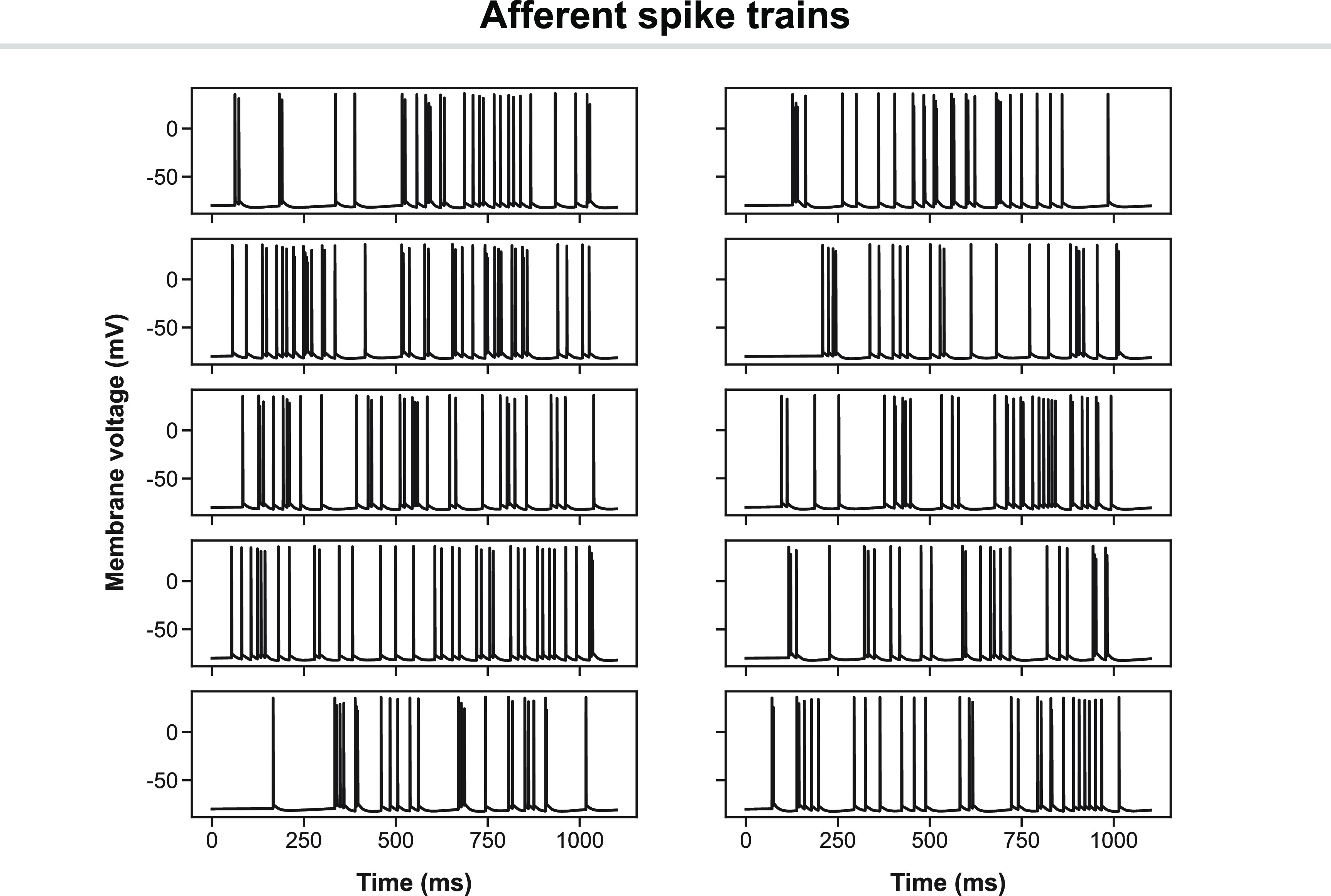
We generated ten random spike trains to model ongoing activity in afferent fibers. We generated spike trains as homogeneous Poisson processes with a mean rate of 30 spikes per second. All analyses used the same ten spike trains depicted in the figure.

For these analyses, we introduced spontaneous ongoing spike trains in the form of homogeneous Poisson processes with a mean rate of 30 spikes per second. We generated ten random spike trains with these parameters (figure 5). We used the same ten randomly generated spike trains across all trials. Thirty spikes per second is in the range for spontaneous firing of human large-diameter myelinated afferents [40, 41] and various classes of C-fibers [42] that have been observed in microneurography experiments. We performed this analysis for both C-fibers and DC A*β*-fibers. We modeled A*β*-fibers using a previously developed adaptation of the McIntyre-Richardson-Grill (MRG) axon model, which is the gold standard for modeling mammalian axons [43, 44]. Briefly, the MRG model is a double-cable axon with finite myelin impedance that permits both intra-axonal and peri-axonal (i.e. submyelin) current flow. Nodes of Ranvier contain active fast sodium, persistent sodium, and slow potassium conductances, as well as passive leak and capacitive currents, whereas the internodal compartments contain only passive leak and capacitive conductances.

We assessed the effects of the various SCS waveforms on spike timing by comparing simulations with and without concomitant SCS. We quantified dissimilarities between spike trains using the Victor–Purpura (VP) distance [45]. This metric calculates the minimum ‘cost’ to transform one spike train into another using three basic operations: spike insertion (cost = 1), spike deletion (cost = 1), and spike shifting (cost = *q*|Δ*t*|, where Δ*t* is the time-shift interval and *q* is the cost of shifting per time unit). Here, we set *q* = 1. We standardized the dissimilarities by first finding the average pairwise VP distance between the ten randomly generated spontaneous spike trains. We then divided all further VP distances by this average value. Thus, a value close to 1 suggests dissimilarity that is close to the average dissimilarity score of random spike trains generated by an identical homogeneous Poisson process.

### 2.5. Stochastic ion channel gating in DC fibers

Typically, neuron models are parameterized using deterministic sets of differential equations, often derived from averaging a collection of recordings. However, ion channel gating is inherently stochastic in nature, and the state of an ion channel (i.e. whether it is open or closed) fluctuates over time in a probabilistic fashion [46]. Importantly, the impact of these fluctuations will be larger in smaller fibers as they have fewer ion channels, thus magnifying the impact of fluctuations in individual channels [47]. This stochastic gating can affect both activation threshold as well as firing patterns in activated fibers, potentially generating pseudo-spontaneous and asynchronous firing [30]. For this reason, the interaction of stochastic ion channel properties with novel high-frequency stimulation paradigms has been proposed as a potential mechanism underlying paresthesia-free analgesia [31].

As previously described, we modeled A*β*-fibers using the MRG axon model. We utilized a previously developed diffusion approximation approach to incorporate stochastic ion channel properties into the MRG model [48]. In short, this approach uses stochastic differential equations to model the fraction of channels in each state, which is considerably more computationally efficient than methods utilizing Markov Chains. The time evolution of the fraction of channels in each state is modeled by the combination of a deterministic component, which use the same voltage-dependent rate constants in the traditional model, and a stochastic component, which includes independent Gaussian noise processes to introduce stochasticity. The precise details of this approach, and its mathematical derivation, are provided by Orio and Soudry [48].

We evaluated the effects of stochastic ion channels on superficial DC fibers. Specifically, we placed modeled fibers at the midline of the DC white matter and 100 *μ*m ventral to the surface of the spinal cord. We modeled fibers using a modified version of the MRG axon model, developed in a previous study to improve the dynamics of the potassium conductance [43, 44]. Channel densities were identical to those in the original MRG model: 2000 channels per *μ*m^2^ for nodal fast sodium and 100 channels per *μ*m^2^ for nodal potassium [44]. We evaluated thresholds for fiber diameters between 5.7 and 11.5 *μ*m by running 50 simulations for each diameter and stimulation waveform, each with a unique random seed. Additionally, to evaluate the effect of stochasticity on firing patterns, we also ran simulations at an amplitude 10% greater than the deterministic threshold determined for each SCS stimulation waveform and fiber diameter (i.e. 10% greater than the amplitude necessary to generate at least one action potential in the standard fiber model). We ran all simulations with a time step of 1 *μ*s.

### 2.6. Local cell polarization

**Figure 6.**
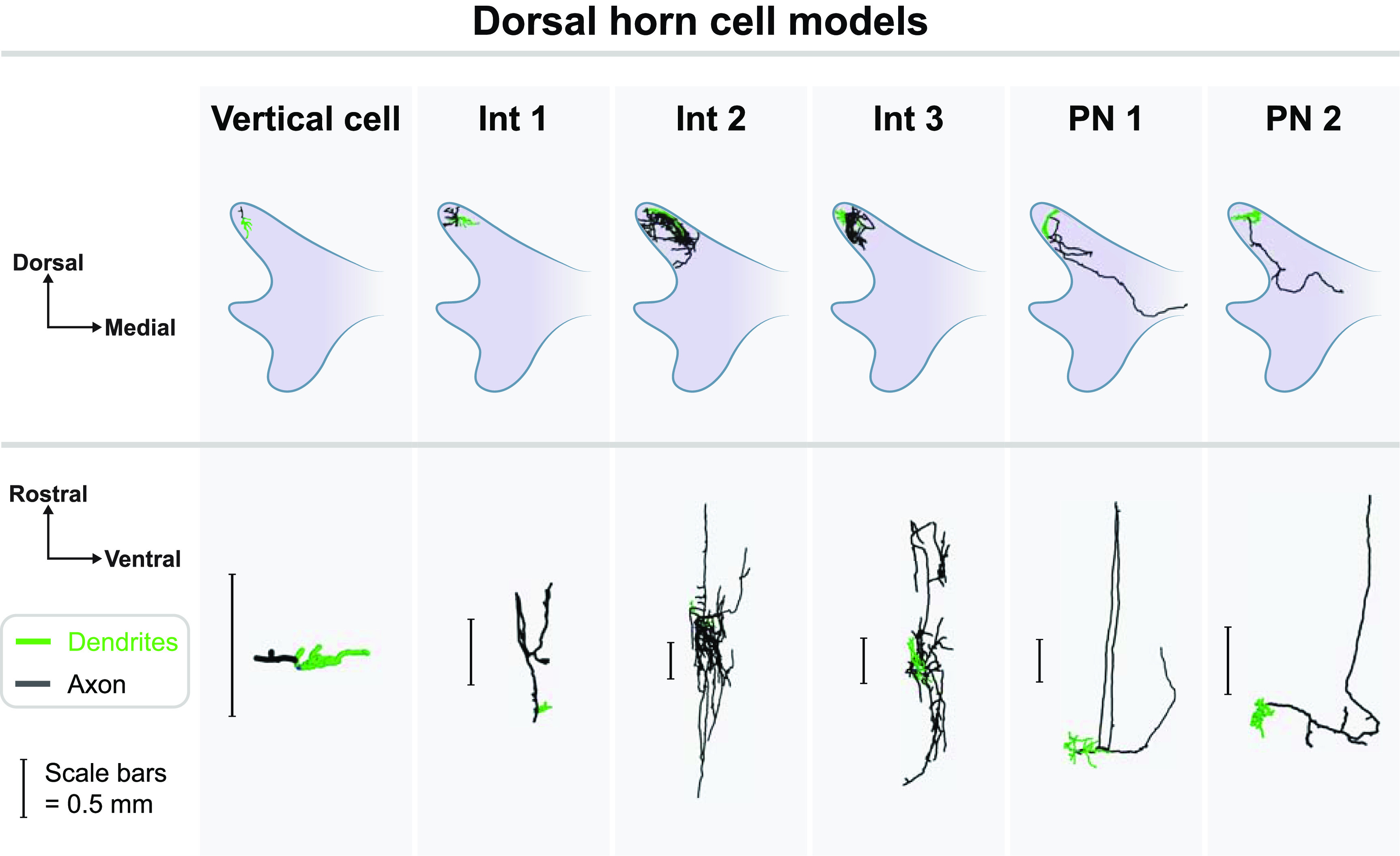
Local cell models in the dorsal horn. Top and bottom rows show axial and sagittal views, respectively, of neurons within the dorsal horn. Dendrites are shown in green and axons in black. The vertical cell is a simplified version of Interneuron 1 (Int 1). All other cell models were based on experimental reconstructions. Scale bars in the sagittal view each correspond to 0.5 mm for the adjacent neuron. Int: interneuron, PN: projection neuron.

**Figure 7.**
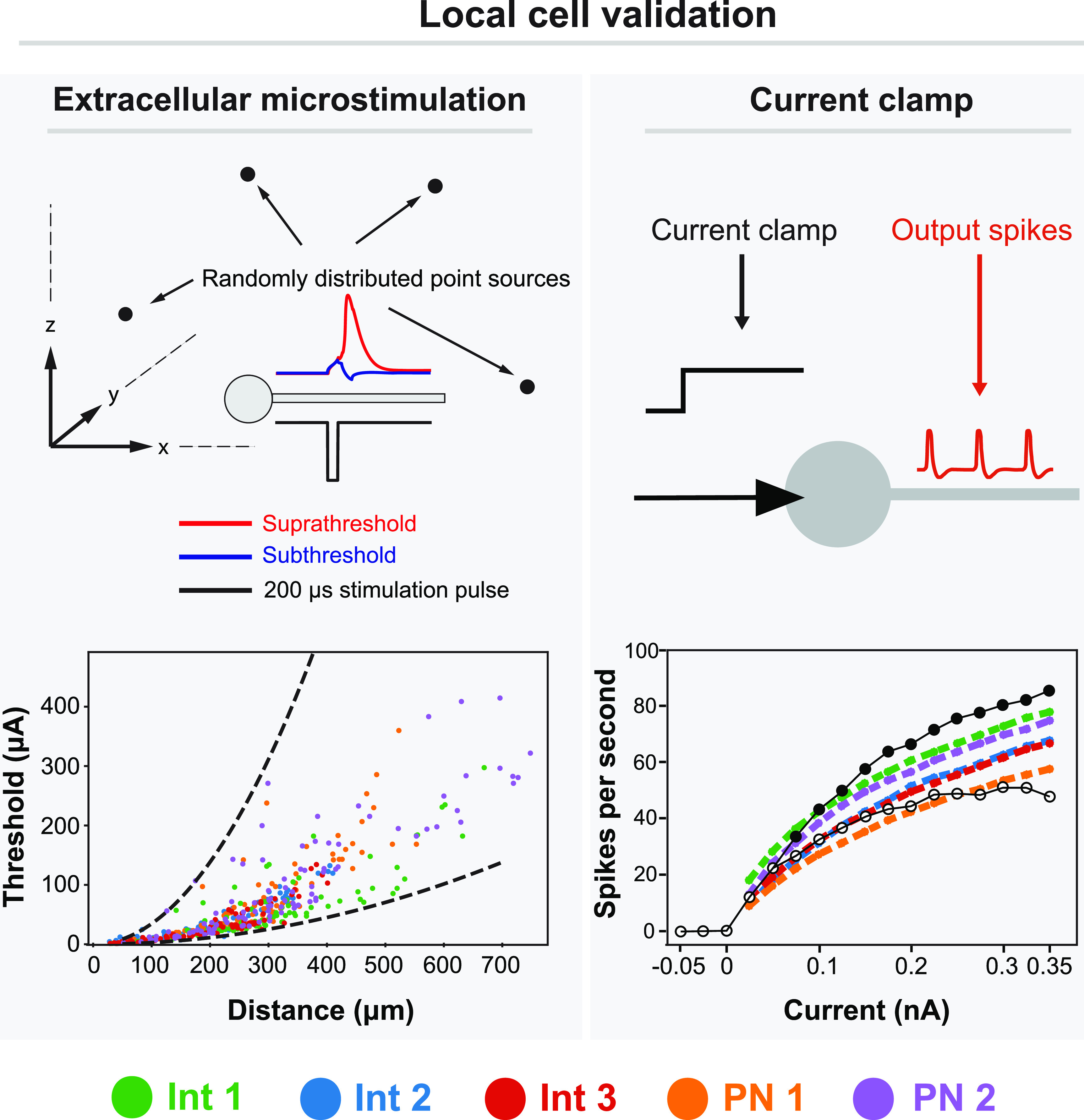
Validation of local cell models. Left: activation thresholds during extracellular microstimulation with a 200 *μ*s cathodic pulse. We randomly placed 100 point-source electrodes in the vicinity of each of the five neuron models. As shown in the bottom left plot, we calculated the activation threshold (ordinate) for each neuron model (color coded) and the distance between the corresponding point-source electrode and site of activation (abscissa). The dashed black lines correspond to the minimum and maximum experimental values given by Stoney *et al* [53]. Right: firing rate of each of the five model neurons during simulated current clamp conditions compared to experimental recordings of dorsal horn tonic firing neurons by Ruscheweyh and Sandkühler [52] (open circle = lamina I neurons, filled circles = deeper neurons). Microstimulation thresholds and current clamp results are color-coded for each of the five model neurons (Int: interneuron, PN: projection neuron), with the names corresponding to the model neurons shown in figure 6.

We developed models of five cells within the superficial dorsal horn based on morphological reconstructions of superficial dorsal horn neurons (figure 6). Three of these neurons were interneurons and two were projection neurons. We downloaded these morphologies from Neuromorpho (Neuromorpho ID numbers NMO_61486, NMO_34018, NMO_34025, NMO_34017, NMO_61481) [49–51]. We validated the cell models by comparing their responses to injected current clamp recordings [52] and to extracellular microstimulation [53] (figure 7). For extracellular microstimulation, we positioned 100 point sources randomly in three-dimensional space around the neurons and calculated the activation threshold for a 200 *μ*s cathodic pulse [54]. Time constants for these models ranged from 34.4 to 43.2 ms, which is in excellent agreement with the 40 ± 5 ms reported by Prescott and De Koninck for tonic-firing lamina I neurons [55]. We also developed a vertical cell model, which is a class of neurons residing in lamina II of the superficial dorsal horn that have been implicated in pain processing [56]. These neurons are characterized by their ventrally projecting dendritic arbor. To generate this model, we simplified the axonal and dendritic structure of one of the interneuron models (Interneuron 1 in figure 6), and then re-oriented the cell to have a dorsally-directed axon and ventrally-directed dendritic arbor.

All local neuron models had identical biophysical properties and thus varied solely in morphology. Axons were myelinated following the algorithm described by Aberra *et al* [54] and myelinated compartments had a specific membrane capacitance of 0.02 *μ*F cm^−2^ and membrane resistance of 1.25 MΩ cm^2^. Unmyelinated neuron compartments had a membrane capacitance of 0.85 *μ*F cm^−2^ and membrane conductivity of 1.8 × 10^−5^ S cm^−2^ [57]. Active currents were fast sodium and delayed rectifier potassium, which are sufficient to generate tonic firing patterns found in superficial dorsal horn neurons [58]. Sodium conductances were 1.8, 0.008, and 0.008 S cm^−2^, and potassium conductances were 0.3, 0.0043, and 0.1 S cm^−2^ in the nodes of Ranvier, soma, and dendrites, respectively. Axial resistance was uniformly 200 Ω cm.

We positioned the model neurons in the superficial dorsal horn. We shifted the cell models rostro-caudally in 500 *μ*m increments from 5 mm caudal to the center of the anode to 5 mm rostral to center of the cathode. For each compartment in the neuron models, we calculated the maximum polarization from the resting membrane potential during each stimulation waveform in response to a 1 mA stimulus. Clinically, the various SCS waveforms can use different amplitudes. For example, clinical ranges for burst and 10 kHz SCS have been given as 0.05–1.6 and 1–5 mA, respectively, and conventional paresthesia-based SCS can use a broad range depending on patient preference [15, 20]. Given uncertainties in paresthesia-threshold for each waveform, using a fixed amplitude allows for direct comparison across waveforms, and subthreshold results can be scaled linearly to estimate the response at higher stimulation amplitudes.

## 3. Results

### 3.1. C-fibers are not activated by SCS

We calculated activation thresholds for a C-fiber model as the stimulation amplitude necessary to generate at least one action potential in the fiber during SCS. For all of the SCS waveforms, the activation thresholds for C-fibers were far beyond those stimulation amplitudes necessary to activate myelinated DC A*β*-fibers. We found that burst SCS had the lowest thresholds, followed (in order) by conventional SCS, 1 kHz SCS, and then 10 kHz SCS. We performed a sensitivity analysis to C-fiber rostro-caudal level by placing the C-fiber in several adjacent dorsal rootlets. Of all the C-fiber positions and SCS waveforms tested, the lowest observed threshold for a C-fiber was ∼60 mA during burst SCS, more than an order of magnitude higher than the amplitude necessary to activate the myelinated afferents underlying paresthesia. No other form of SCS activated C-fibers at 100 mA, which was the maximum amplitude tested and well beyond the maximum amplitudes employed clinically. These data provide strong evidence that C-fibers are not activated by clinical SCS.

### 3.2. SCS modulated spike timing in Aβ-fibers at supra-threshold amplitudes, and did not modulate spike timing in C-fibers

We evaluated the effects of conventional, burst, 1 kHz, and 10 kHz SCS on spike timing in A*β*-fibers as well as in C-fibers. We placed the A*β*-fiber superficially in the DC white matter, 100 *μ*m ventral to the surface and at the mediolateral midline of the spinal cord. We modeled the response of a single 10 *μ*m fiber, which corresponds to the upper range of fiber diameters found in the DCs [59]. We then calculated the activation threshold for this model fiber to generate at least one action potential. The activation thresholds were 1.92, 0.96, 2.20, and 8.15 mA for conventional, burst, 1 kHz, and 10 kHz SCS, respectively.

We then evaluated the effects of spike timing on fibers with ongoing spontaneous activity (10 separate iterations of a 30 Hz homogeneous Poisson process) during SCS with the stimulation amplitude set to 0.05 mA below the activation threshold for each waveform. We quantified dissimilarity between spike trains using the VP distance, which we standardized so that a value close to 1 suggests the dissimilarity is close to the average dissimilarity between the ten randomly generated input spike trains (see materials and methods).

For subthreshold stimulation (i.e. SCS amplitude 0.05 mA below the amplitude necessary to generate an action potential), we found that spike timing modulation was negligible. For conventional, burst, 1 kHz, and 10 kHz SCS, the average standardized VP distances (mean ± SD) were 0.002 ± 0.005, 0.004 ± 0.007, 0.011 ± 0.003, and 0.017 ± 0.024, respectively (table 1).

**Table 1.** Average standardized VP distances between spontaneous spike trains with and without applying simultaneous SCS. Data are given as mean ± SD.

	0.05 mA below threshold	0.1 mA above threshold	0.3 mA above threshold	0.5 mA above threshold
Conventional	<0.01	0.04 ± 0.02	0.17 ± 0.04	0.40 ± 0.02
Burst	<0.01	0.10 ± 0.04	0.53 ± 0.10	1.25 ± 0.13
1 kHz	0.01 ± 0.00	0.10 ± 0.06	0.44 ± 0.15	1.58 ± 0.15
10 kHz	0.02 ± 0.02	0.06 ± 0.04	0.07 ± 0.04	0.13 ± 0.05

**Figure 8.**
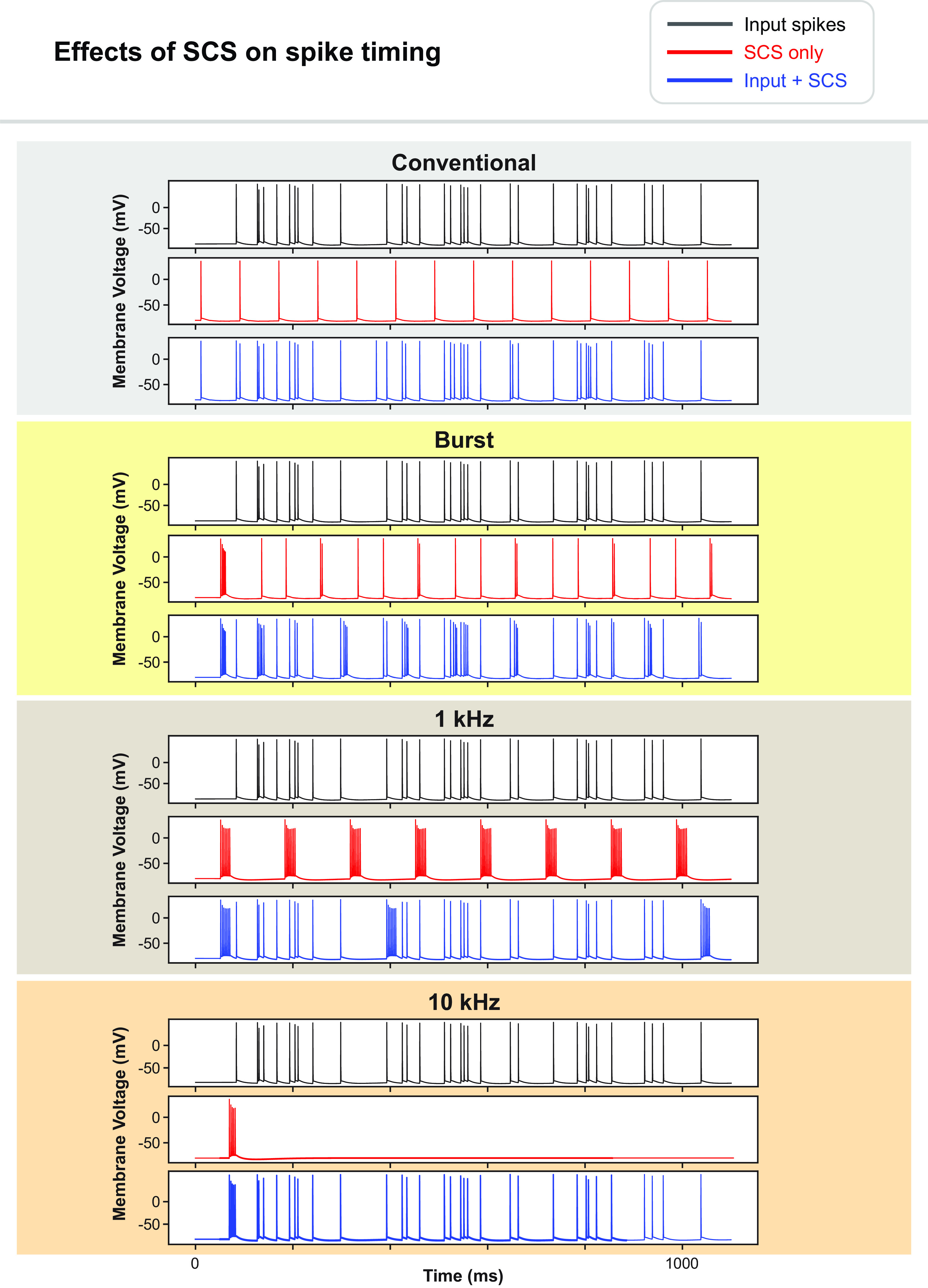
Representative example of the effects of suprathreshold SCS (0.3 mA above activation threshold) on spike timing during conventional, burst, 1 kHz, and 10 kHz SCS. For each SCS waveform, three membrane voltage traces are provided: (top) the ongoing spontaneous input within the fiber. (Middle) The response of the fiber to SCS with no ongoing spontaneous input. (Bottom) The response of the fiber when the stimulation is provided while there is simultaneous spontaneous activity.

Next, we investigated the effects on spike timing when the amplitude was increased to slightly above threshold, as it is possible that SCS may activate DC fibers without concomitant paresthesia [60]. Thus, the amplitude is above the threshold for activating the specific fiber under consideration, but not necessarily strong enough to generate a perceptible sensation. We repeated the same analysis as in the subthreshold case, but at amplitudes 0.1, 0.3, and 0.5 mA above the activation threshold for each of the SCS waveforms. For context, typical clinically effective amplitude ranges for burst and 10 kHz SCS have been given as 0.05–1.6 (mean ∼ 0.6 mA) and 1–5 mA, respectively [15, 20]. In this case, appreciable differences in spike timings were observed compared to the spontaneous input spike trains (summarized in table 1). We observed two clear trends. First, for the stimulation amplitudes 0.1, 0.3, and 0.5 mA above the activation threshold, this dissimilarity almost always included an increase in the number of action potentials compared to the input train (30/30, 30/30, 28/30, 24/30 for conventional, burst, 1 kHz, and 10 kHz SCS, respectively). For the remaining small number of cases, the number of spikes remained the same (0/30, 0/30, 2/30, 6/30 for conventional, burst, 1 kHz, and 10 kHz SCS, respectively). Second, burst and 1 kHz SCS had similarly large effects on spike trains, whereas conventional and 10 kHz SCS produced notably smaller standardized VP distances at each amplitude. A representative example for stimulation 0.3 mA above threshold is provided in figure 8.

Finally, it has been proposed that novel SCS waveforms can desynchronize nociceptive signaling in C-fibers. To this end, we repeated the above analysis in the C-fiber models. We placed the C-fiber in the dorsal rootlet and Lissauer’s tract, which is superficial to the dorsal horn and adjacent to the DCs. We performed sensitivity analysis by shifting the fiber into the adjacent dorsal rootlets to ensure that results were consistent across rootlets and rostro-caudal position. For each SCS waveform, we performed this analysis with an amplitude of 120% the necessary amplitude to activate the 10 *μ*m DC fiber. Alterations in spike timing were negligible (standardized VP distance <0.01).

### 3.3. Stochastic ion channel behavior affected firing patterns, but not activation thresholds

**Figure 9.**
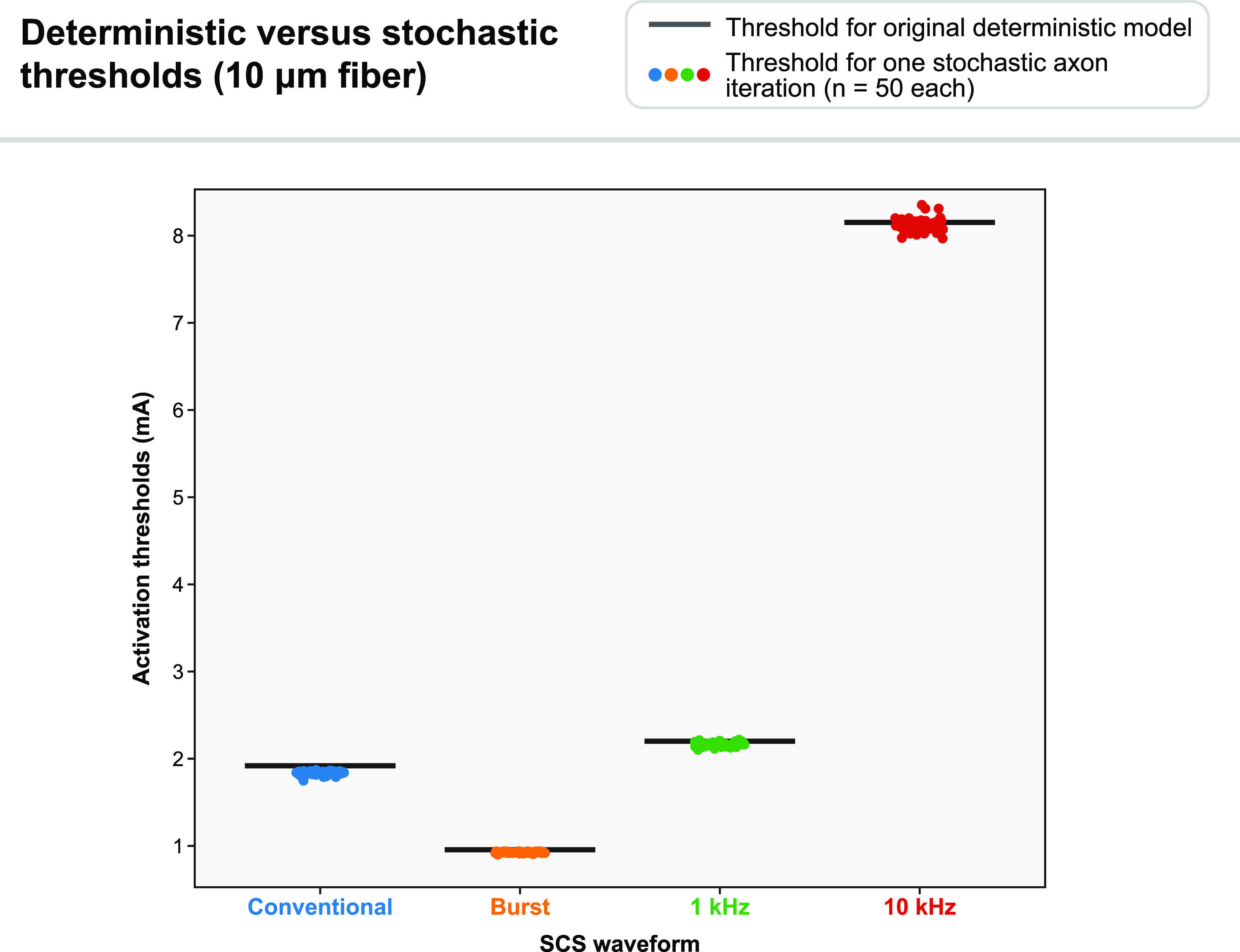
Deterministic (black lines) versus stochastic (circles) model thresholds for a superficially positioned 10 *μ*m DC fiber. For each SCS waveform, we found thresholds for 50 randomly seeded stochastic models.

We incorporated stochastic ion channel behaviors into superficial DC fibers between 5.7 and 11.5 *μ*m in diameter (50 fibers per diameter). Overall, the activation thresholds were highly similar to those found in the deterministic model, with a tendency for stochastic models to have slightly lower thresholds. Differences between the stochastic model and the deterministic model were negligible for the largest-diameter fibers (e.g. fibers ⩾ 8.7 *μ*m). Smaller-diameter fibers had slightly larger variation in thresholds, but the order of recruitment was always the same as for the deterministic model (table 2 and figure 9).

**Table 2.** Activation thresholds for a superficial DC fiber using the deterministic (D) and stochastic (S) models. Thresholds are in mA. For the stochastic models, thresholds are given as mean ± SD.

	5.7 *µ*m		7.3 *µ*m		8.7 *µ*m		10.0 *µ*m		11.5 *µ*m	
Axon diameter	D	S	D	S	D	S	D	S	D	S
Conventional	5.60	5.22 (±0.09)	3.27	3.09 (±0.04)	2.34	2.23 (±0.03)	1.92	1.83 (±0.02)	1.66	1.59 (±0.01)
Burst	2.67	2.43 (±0.04)	1.59	1.52 (±0.02)	1.17	1.12 (±0.02)	0.96	0.92 (±0.01)	0.84	0.81 (±0.01)
1-kHz	6.88	6.61 (±0.11)	3.90	3.81 (±0.04)	2.71	2.65 (±0.03)	2.20	2.16 (±0.02)	1.88	1.85 (±0.02)
10-kHz	27.49	27.25 (±0.48)	15.43	15.37 (±0.20)	10.32	10.29 (±0.11)	8.15	8.12 (±0.08)	6.72	6.70 (±0.06)

**Figure 10.**
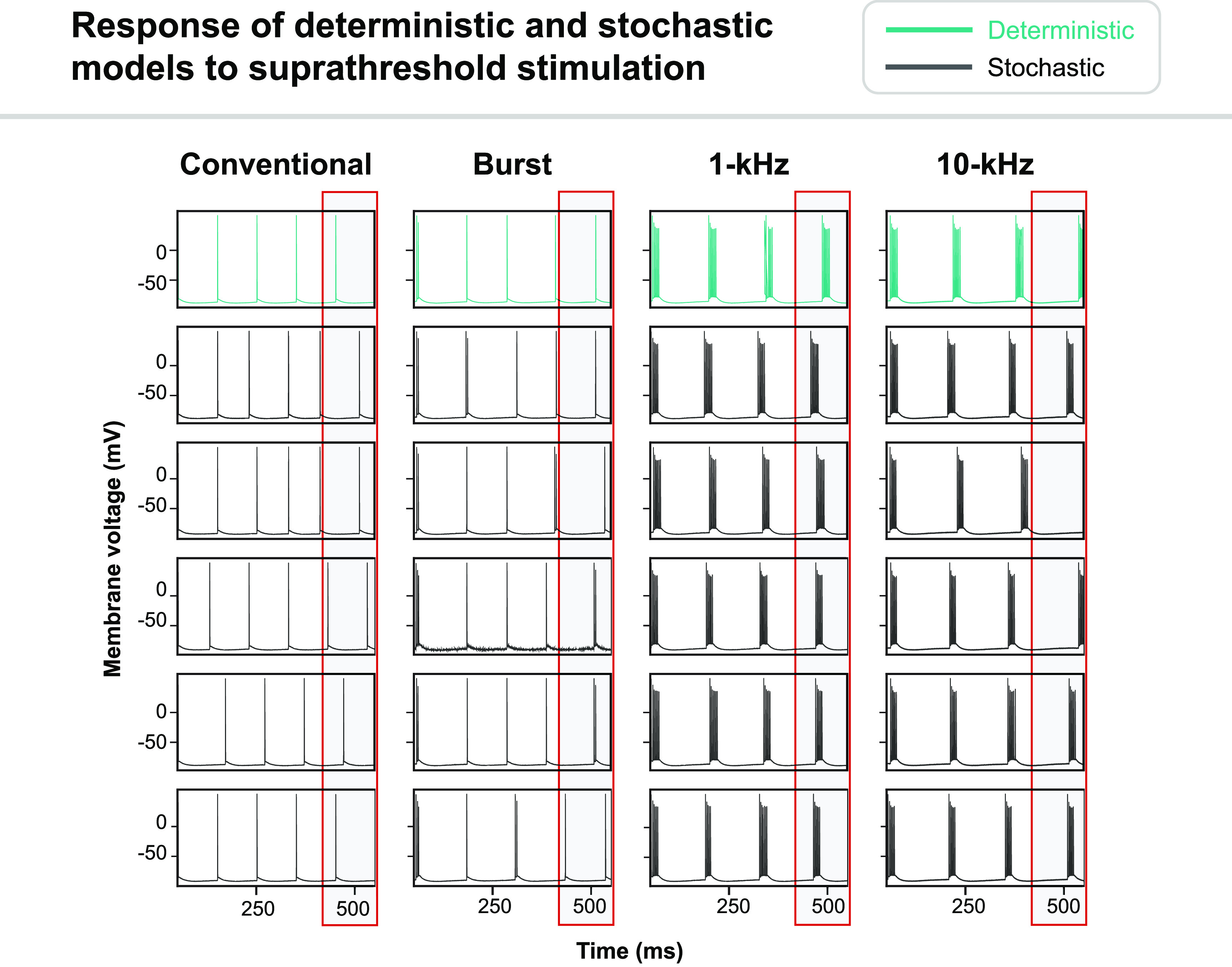
Comparison of the response of a 10 *μ*m DC fiber to conventional, burst, 1 kHz, and 10 kHz SCS using deterministic (green) and stochastic (black) ion channel models. The stimulation amplitude was 10% above the activation threshold for the deterministic model. For each SCS waveform, we randomly selected five stochastic simulations represented by each row in the plot above. Boxes at the end of each simulation highlight differences in spike timing between the deterministic and stochastic models.

Next, we evaluated how incorporating stochastic ion channel gating affected firing properties by modeling 10 *μ*m DC fibers with a stimulation amplitude 10% greater than the deterministic threshold. We found that firing patterns were qualitatively similar for stochastic models compared to the original deterministic model, but we observed slight differences in spike timing and the number of spikes (figure 10).

### 3.4. Local cell polarization was largest in the axon

Direct modulation of rostrally-oriented dorsal horn neurons has been proposed as a mechanism of action for several SCS paradigms [18, 27, 28]. To test this hypothesis, we generated models of five neurons within the dorsal horn (three interneurons, two projection neurons) based upon previously published morphological reconstructions, as well as a vertical cell model with a dorsally-oriented axon (see materials and methods). Neurons spanned from 5 mm caudal to the center of the anode to 5 mm rostral to the center of the cathode in 500 *μ*m increments (total of 37 locations).

In agreement with previous work [61], we found that activation thresholds for all of the dorsal horn neurons were well above those for DC fibers for all SCS waveforms (data not shown). However, a neuron does not need to be directly activated to have its behavior modulated by the stimulation [62, 63], and thus we assessed membrane polarization through the soma, dendrites, and axonal arbor to investigate whether a neuron was likely to experience subthreshold modulation (e.g. activation due to spatiotemporal summation of subthreshold currents, increased excitability to synaptic input, or altered presynaptic transmitter release). For each waveform, we evaluated the maximum depolarization (and hyperpolarization) throughout the cell during stimulation at 1 mA (responses can be scaled to estimate the response at a higher stimulation amplitude).

Overall, the polarization magnitudes were small, and were varied across the different neurons, which affirms that cellular morphology is important to consider for neurostimulation applications (table 3). We found that in all cases, burst SCS produced the strongest polarization, whereas 10 kHz SCS always produced the smallest polarization. Importantly, polarizations were always largest in the axon and negligibly small in the soma, whereas dendritic polarizations were typically non-negligible but substantially smaller than in the axon. The largest axonal depolarizations during a unit amplitude (1 mA) stimulus for conventional, burst, 1 kHz, and 10 kHz SCS, were 0.71, 1.09, 0.6, and 0.22 mV, respectively (all in the same neuron; Interneuron 1 in figure 6). For dendrites, maximum depolarizations (across all neuron morphologies and rostrocaudal positions) were approximately 0.2 mA for burst SCS, 0.1 mA for conventional SCS, 0.1 mA for 1 kHz SCS, and 0.05 mA for 10 kHz SCS.

**Table 3.** Maximum depolarizations in mV in the different compartments during the various forms of SCS for each neuron. Note, these are the overall maximum depolarizations observed across all rostrocaudal levels. C = conventional, B = burst, 1 K = 1 kHz, 10 K = 10 kHz.

	Axon				Dendrites				Soma			
	C	B	1 K	10 K	C	B	1 K	10 K	C	B	1 K	10 K
Vertical cell	0.43	0.47	0.41	0.24	0.07	0.11	0.06	0.03	0.03	0.06	0.04	0.01
Interneuron 1	0.71	1.02	0.60	0.22	0.09	0.19	0.07	0.02	0.02	0.05	0.03	0.01
Interneuron 2	0.39	0.68	0.31	0.10	0.09	0.12	0.07	0.03	0.04	0.07	0.03	0.01
Interneuron 3	0.30	0.73	0.43	0.12	0.09	0.19	0.07	0.03	<0.01	0.02	<0.01	<0.01
Projection Neuron 1	0.20	0.38	0.16	0.09	0.10	0.18	0.08	0.03	0.01	0.02	0.01	<0.01
Projection Neuron 2	0.28	0.65	0.22	0.08	0.12	0.17	0.10	0.06	0.02	0.05	0.02	0.01

**Figure 11.**
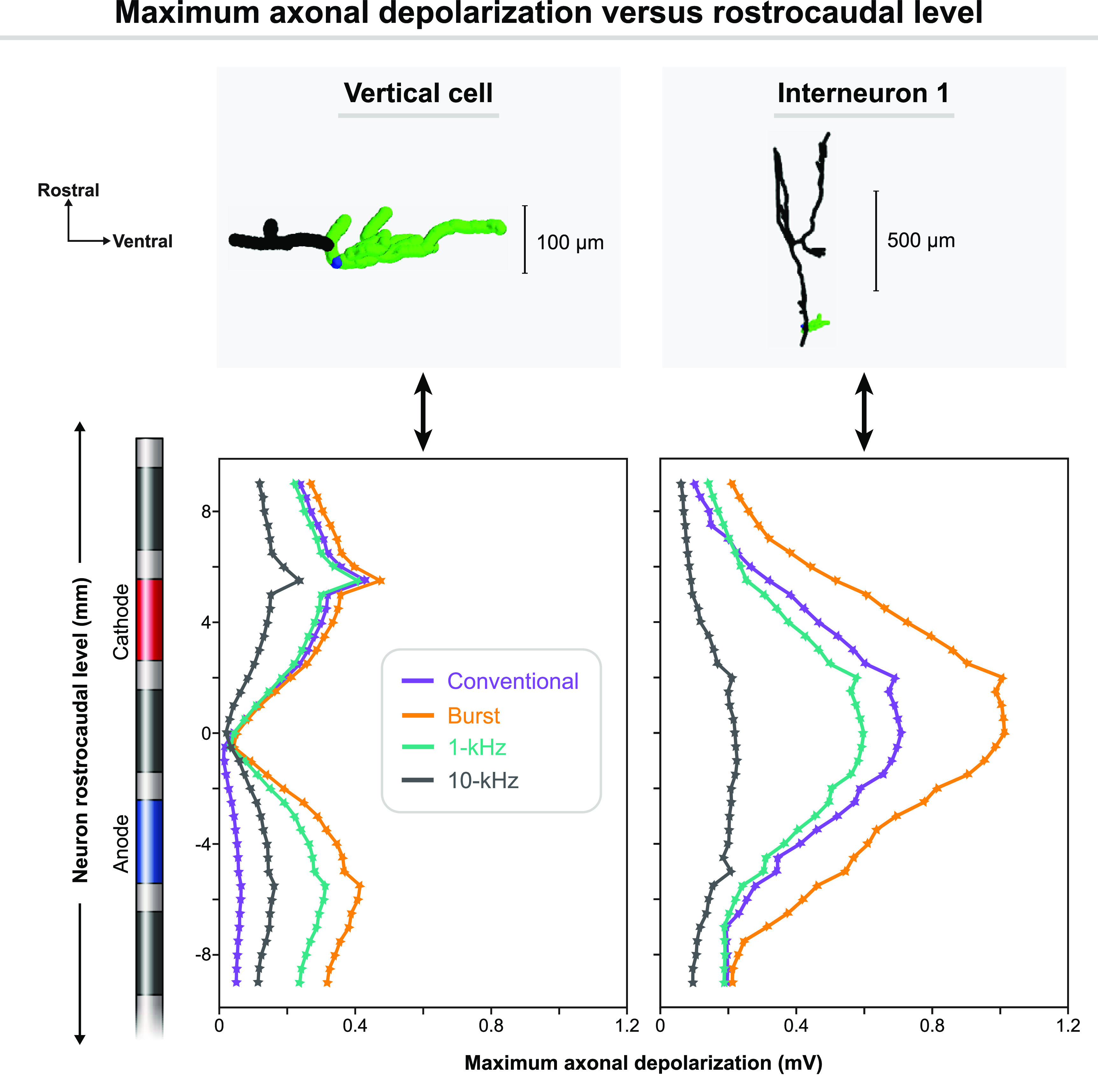
Effects of rostrocaudal position on maximum axonal depolarization for (left) the vertical cell and (right) a rostrocaudally oriented interneuron. The interneuron corresponds to Interneuron 1 in figure 6, which was the most strongly polarized of all neuron models. Different colored lines correspond to the different SCS waveforms. Filled markers indicate the maximal depolarization from rest within the axon at a given rostrocaudal level. Simulations were sampled at 500 *μ*m increments from 5 mm caudal to the center of the anode to 5 mm rostral to the center of the cathode.

Finally, we found that local cell polarization was strongly dependent on rostrocaudal position as well as cellular morphology and orientation (figure 11). For neurons with rostrocaudal orientations (e.g. Interneurons 1–3 in figure 6), polarization in the axon was strongest approximately midway between the two active electrodes. On the other hand, the dorsally-directed vertical cell axon was maximally depolarized at rostrocaudal levels near the outer edges of the active contacts.

## 4. Discussion

Several new forms of SCS produce analgesia without producing the concomitant paresthesia inherent to conventional stimulation. These therapies offer promising options for patients who do not tolerate these paresthesias and may even produce superior pain relief compared to conventional SCS [8, 16, 37]. Still, little is known about the neurophysiological effects and underlying mechanisms of action of these paradigms. A better mechanistic understanding of these stimulation waveforms will facilitate the development of optimized stimulation settings to maximize pain relief, tailored to an individual patient’s condition, while minimizing power consumption and prolonging battery life. Numerous hypotheses have been proposed to underlie the effects of these novel paradigms. Particularly common and well-received hypotheses include selective activation of anti-nociceptive C-fibers (thereby affecting the medial pain pathway), desynchronization of afferent signaling, pseudo-spontaneous activation of DC fibers, and direct modulation of dorsal horn neurons [18, 20, 26–28]. Unfortunately, direct experimental testing of these hypotheses is often infeasible, and anatomical differences between humans and animal models complicate direct translation of the available preclinical evidence. Therefore, in this study, we used a computational modeling approach to investigate the neurophysiological effects and potential mechanisms of action of three subparesthetic SCS paradigms (burst, 1 kHz, and 10 kHz SCS), as well as conventional paresthesia-based SCS.

### 4.1. C-fibers are not activated or modulated by SCS

Activation thresholds for C-fibers were well beyond the activation thresholds of A*β*-fibers in the DCs, refuting the notion that C-fibers are selectively activated by subparesthetic SCS waveforms. Similarly, the timing of ongoing spikes in C-fibers were not affected by any form of SCS. One consideration is that these waveforms have been suggested to activate non-noxious C-fibers that convey pleasant touch [64], whereas we utilized a model derived for a nociceptive C-fiber [38]. However, while differences in their biophysical properties could affect their response to stimulation, it is unlikely that these differences would reduce thresholds at least an order of magnitude to be comparable to A*β* fibers, as their small diameter and unmyelinated structure strongly oppose activation by extracellular stimulation.

### 4.2. Local cell membrane polarization is primarily in the axon

We analyzed membrane polarization during SCS for six models of local dorsal horn cells. These were three rostrocaudally oriented interneurons, two projection neurons, and one dorsoventrally oriented interneuron (a vertical cell). For all neurons, activation thresholds were notably higher than those for DC fibers, suggesting that it is unlikely that these neurons are activated during clinical SCS (data not shown).

It has been hypothesized that SCS preferentially activates or increases excitability in the dendrites of local dorsal horn neurons [27, 28]. Our results demonstrated that dendrites were weakly polarized by unit 1 mA stimulation, with an overall maximum depolarization of ∼0.2 mV during burst SCS, and maximum polarizations were less than or equal to 0.1 mV during 1 kHz and 10 kHz SCS. Clinical SCS is often delivered at higher amplitudes than 1 mA and these depolarizations would increase at these higher stimulation amplitudes. However, maximum depolarization in the axon was always at least twice as large as the depolarization in the dendrites, and often at least four or five times larger. Additionally, we note that maximum depolarization in the dendrites of the vertical cell, which are directed ventrally, were comparable to those for the other neurons, suggesting that bipolar stimulation will not preferentially modulate dendrites in rostrocaudally oriented neurons. From these results, we conclude that a presynaptic mechanism of action (e.g. altering neurotransmitter release) is more likely than effects on somatodendritic processing. It is worth noting that primary afferent collaterals will also produce rostrally oriented arborizations in the superficial dorsal horn [65, 66]. Thus, any presynaptic effects on local cells could plausibly be mirrored in these afferent fiber terminals, and these effects warrant consideration in future studies.

We also observed a strong effect of rostrocaudal position on axonal polarization. Specifically, in this bipolar configuration, the strongest axonal polarization for the rostrocaudally oriented cells was approximately at the midpoint between the electrodes. On the other hand, polarization for the dorsally directed vertical cell axon was maximal near the lower and upper borders of the anode and cathode, respectively. This vertical cell polarization was of a similar magnitude to that observed for the other cells. Intuitively, these observations are consistent with the notion that these axon terminals will be most strongly polarized where the electric field parallel to the terminating axon is maximized [67]. Thus, these results demonstrate that modulation of local neurons in the dorsal horn is not necessarily selective for specific neuron orientations, but that different cell morphologies will be modulated simultaneously at different locations in the spinal cord.

### 4.3. Effects of stochastic ion channels

We found that incorporating stochastic ion channel behavior did not alter activation thresholds but did affect firing patterns in DC fibers. Specifically, stochastic ion channels produced slight alterations in the timing and number of spikes, although firing patterns were qualitatively similar (figure 10). Recent experiments have demonstrated that stimulating DC fibers at amplitudes just above activation threshold will produce asynchronous firing responses [68]. While deterministic fiber models provide qualitatively accurate approximations of the response to SCS, incorporating the stochastic ion channel properties produced results that demonstrated fluctuations in spike timing that more closely resemble experimental recordings [60, 68].

These fluctuations have several important clinical and technical implications. First, Gilbert and colleagues demonstrated *in silico* that asynchronous firing patterns more effectively reduced the output of the dorsal horn pain processing network than synchronous DC fiber activation [68]. This result highlights the need to understand realistic DC fiber firing patterns during SCS to better maximize pain relief, which our results demonstrate are noticeably affected by including stochasticity. Next, recent evidence suggests that paresthesia may rely on the synchrony of DC fiber activation [60]. Thus, asynchronous firing due to these stochastic ion channel properties could increase the paresthesia perception amplitude, and future waveforms could be designed to exploit these properties to reduce synchronization and thereby increase the therapeutic range of stimulation at subparesthetic levels.

### 4.4. Comparison of modeling results with existing experimental evidence

Several experimental studies have evaluated the effects of novel stimulation paradigms on DC fibers or dorsal horn neurons. In the DCs, Crosby *et al* extracellularly recorded the response of single DC fibers undergoing conventional 50 Hz SCS as well as 1, 5, 10, and 20 kHz stimulation in rats [69]. In line with our modeling results that conventional SCS had a lower activation threshold than 1 kHz SCS, which in turn had a notably lower activation threshold than 10 kHz stimulation (table 2 and figure 9), Crosby *et al* found that motor thresholds followed this same pattern, although direct comparison between our modeling results and their study is complicated by the use of different pulse widths [69]. Interestingly, they also found that kilohertz-frequency SCS tended to produce accommodation, in which activated axons would cease firing after continued application of the stimulus. This effect is not accounted for in our model, in which axons could continue to fire indefinitely in regular patterns, and it is a current limitation in the current gold standard MRG-based modeling approach. However, our results do predict that kilohertz-frequency SCS will produce bursts of action potentials at suprathreshold amplitudes (figure 8), which agrees with recent recordings during kilohertz-frequency stimulation by Sagalajev *et al* [60].

In the rat dorsal horn, Lee *et al* found that low amplitude 10 kHz SCS selectively increased firing rates of putative inhibitory interneurons while not affecting excitatory interneurons, whereas 1 kHz and 5 kHz SCS did not induce significant firing in either cell type [18]. In a follow-up study, Lee and colleagues reported that 10 kHz SCS produced greater increases in the firing of inhibitory interneurons relative to burst SCS, while simultaneously driving less firing in excitatory neurons than burst SCS [70]. In contrast, Kuo *et al* recently found that 50 Hz, 1.2 kHz, and 10 kHz SCS could modulate activity in rat dorsal horn neurons below the electrode at an amplitude 40% of motor threshold, which was assumed to be subparesthetic [28]. Importantly, results were heterogeneous across cell types (defined by response to various painful and non-painful mechanical stimuli) and could demonstrate both an increase or decrease in firing rates.

Although differing in results, these studies all found evidence for direct modulation of dorsal horn neurons at amplitudes presumed to be subparesthetic, which disagrees with our analysis that local cells are weakly polarized by SCS, with burst SCS producing the strongest effects and 10 kHz SCS the weakest effects (table 3). One important consideration is the effect of anatomy. These experimental recordings were from the rat lumbar spinal cord, which notably differs from the relevant human anatomy in important factors including cerebrospinal fluid thickness and superficial dorsal horn depth, allowing greater penetration of the electric field into the gray matter [71], as well as differences in DC fiber diameters and dimensions of preclinical versus clinical SCS leads. Furthermore, it is impossible to verify that these amplitudes are subparesthetic in rodents, and observed effects may be driven trans-synaptically by activation of DC fibers. With that said, both our modeling results and the preclinical evidence highlight the possibility for heterogeneous effects on dorsal horn neurons. Namely, we found that both cellular morphology and rosto-caudal position strongly influenced neuronal polarization during all SCS waveforms (table 3 and figure 11). This result may help explain the variable results across experimental studies and the observed variability in responses across various neuronal subtypes.

### 4.5. Limitations and future directions

The results presented in this study further elucidate the biophysical effects of various clinically relevant SCS waveforms. However, there are some limitations to our approach that should be considered. First, we modeled the neural response to a single percutaneous electrode array using a bipolar stimulation configuration, whereas modern SCS systems often employ more than one electrode array and/or complex multipolar stimulation configurations [12]. Still, bipolar stimulation is ubiquitous, and our model represents a reasonable starting point for future work comparing the effects of different configurations on the neural response to SCS [72]. Additionally, our model only considered a single electrode position in a canonical lower thoracic spinal cord. Future work using patient-specific modeling based on subjects’ medical imaging will allow us to account for variations in inter-subject anatomy and electrode placement to confirm that these results are consistent and generalizable [73, 74]. Furthermore, while DC fibers are sensory fibers, we used a motor axon model (i.e. the MRG model) [44]. The MRG model is the gold standard for mammalian axons, but future work developing validated sensory fiber models will be important to understand the importance of considering electrophysiological differences in these fiber classes. For instance, a recent SCS modeling study presented a sensory axon model that reproduces several experimental properties of sensory afferent fibers (and meaningfully differed from the MRG axon model) [75]. Finally, while we are investigating the mechanisms of subparesthetic SCS, very little is known about the precise neural activation profile that generates paresthesia. Thus, we were unable to define a precise stimulation range that corresponds to subparesthetic SCS, which should be considered when interpreting our results. For example, we analyzed our results for the local cell models by comparing depolarizations in the different cellular substructures (i.e. the soma, axon, and dendrites) in response to a unit (1 mA) stimulus, and we found that the axons were notably more depolarized than the soma and dendrites. However, if the perception threshold amplitude is high, it is possible that the soma and dendrites will be sufficiently depolarized to modulate neural behavior. Similarly, we analyzed the effects of suprathreshold stimulation on a single fiber, where threshold was defined as the amplitude necessary to generate an action potential within this fiber. While this biophysical analysis is generalizable to any given axon, it is possible that stimulation could be above threshold for a given axon but still below the necessary amplitude for perceptible sensation. Future work investigating the neural correlates of paresthesia will better inform future studies and clinical SCS practice [60].

## 5. Conclusions

In this study, we utilized an *in silico* approach to evaluate several hypothesized mechanisms of action of conventional, burst, 1 kHz, and 10 kHz SCS. Our results strongly refute the proposal that novel SCS waveforms preferentially activate C-fibers [20]. Additionally, we found that no waveform desynchronized or modulated spike timings in C-fibers, and only substantially did so in A*β*-fibers at suprathreshold amplitudes. Relative to deterministic models, including stochastic ion channel properties into DC fiber models did not appreciably affect activation thresholds but did produce variable and asynchronous firing patterns at suprathreshold amplitudes. Future work will be necessary to understand the effects of these realistic spiking fluctuations on pain relief and paresthesia during SCS. Finally, we found that axons were preferentially polarized during stimulation to a much stronger extent than the soma or dendrites. From this result, we conclude that any SCS-induced modulation of cells within the dorsal horn is likely to be presynaptic in nature (i.e. altered neurotransmitter release). Additionally, we found that cellular morphology and orientation strongly affected both the magnitude of this polarization as well as the rostrocaudal level at which specific cell types were most strongly affected by stimulation. Thus, the heterogeneous cellular populations of the dorsal horn may be differentially modulated at various locations during SCS due to these properties. Overall, the results presented this study help provide a more complete understanding of the neural response to various clinically relevant SCS waveforms.

## Data Availability

The data that support the findings of this study are available upon reasonable request from the authors, while the files to generate the local cell models are openly available at the following URL/DOI: https://github.com/neuromodulation-um/local_cells.
